# Supplementary material for: Advances in Bacteriophage Therapy against Relevant MultiDrug-Resistant Pathogens
Source: Antibiotics (Basel). 2021 Jun 4;10(6):672. doi: 10.3390/antibiotics10060672 (PMC8226639; doi:10.3390/antibiotics10060672)
Supplement: Supplementary file 1 [file antibiotics-10-00672-s001.zip › antibiotics-1232717-supplementary.pdf]

Table S1. Summary of case reports using phage therapy against *A. baumannii* in patients to date.

Age & Gender	Type of Infection	Resistance	Bacteriophage	Phage Therapy	Key Clinical Points & Outcome	References
88-year-old, male	Pneumonia	Carbapenem (only sensitive to tigecycline and polymyxin)	Ab_SZ3 13	Increasing concentrations of nebulized phages every 12 h (except the first 2 doses once daily) during 16 d combined with tigecycline and polymyxin E	Lung function improved gradually. No carbapenem-resistant *A. baumannii* isolated after 6 months	[44]
4 patients from 62 to 81 years old	Pneumonia	Carbapenem	Cocktail containing ϕAb121 and ϕAb124	Six treatments of 2 doses of inhaled phages spaced 1 h	Patients 1 and 2 improved their chest radiographs and discharged hospital. Patient 3 resolved the *A. baumannii* infection but died due to another infection. Patient 4 improved and discharged ICU but died 1 months later of respiratory failure	[45]
42-year-old, male	Poly-microbial bone infection	XDR *A. baumannii* and MDR *K. pneumoniae*	Cocktail containing ϕAbWT21phi3 and ϕKpWT21phi1	Five doses of 1 ml of phages ($5x10^7$ PFU/ml) IV combined with meropenem and colistin followed by six additional days of treatment after one week	*A. baumannii* and *K. pneumoniae* bacteria were not isolated in a follow-up of 8 months	[46]
77-year-old, male	Infection after craniectomy, cerebritis, subdural and epidural empyema	MDR (resistant to all antibiotics, however, some isolates were sensitive to colistin)	Phage cocktail of five phages	96 doses (8 days) of 4 ml of IV phages ($2.14x10^7$ PFU/ml)	Fever and leukocytosis persisted, although were no signs of infection at the craniotomy site. The family decided to withdraw care and the patient died one day afterwards	[47]
68-year-old, male	Necrotizing pancreatitis	MDR	Three phage cocktails: ΦPC (AC4, C1P12, C2P21, C2P24), ΦIV (AB-Navy1, AB-Navy4, AB-Navy71, and AB-Navy97), and ΦIVB (AB-Navy71, AbTP3Φ1)	ΦPC was administered for 18 weeks through percutaneous catheters, ΦIV, during 16 weeks through IV administration, and ΦIVB during 2 weeks via IV ($5x10^9$ PFU). Phage therapy was combined with minocycline, fluconazole and meropenem	Clinical recovery and bacteria eradication	[48]

Abbreviations: ICU, intensive care unit; IV, intravenous; MDR, multi-drug resistant; XDR, extensively drug resistant.

Table S2. Summary of case reports using phage therapy against *K. pneumoniae* in patients from 2019 to date.

Age & Gender	Type of Infection	Resistance	Bacteriophage	Phage Therapy	Key Clinical Points & Outcome	References
66-year-old, male	Multifocal UTI	MDR	ΦJD902 alone and three cocktails: ΦJD902 and ΦJD905; ΦJD905, ΦJD907 and ΦJD908; and ΦJD902, ΦJD905, ΦJD908, and ΦJD910	Only ΦJD902, ΦJD905, ΦJD908, and ΦJD910 cocktail was administered combined with piperacillin/tazobactam	Only after the last cocktail administration, bladder mucosa improved and no recurrence was found after 2 months	[57]
62-year-old, male	Prosthetic joint infection	Strain susceptible to ampicillin-sulbactam, ceftriaxone, ciprofloxacin, meropenem, trimethoprim-sulfamethoxazole, and minocycline	KpJH46ϕ2	40 IV doses of phages (6x10^{10} PFU) combined with oral minocycline	Rapid improvement after 48 h of phage administration and inflammatory markers decreased	[58]
57-year-old, female	UTI	MDR	vB_KpnM_GF	10 ml of oral phages (10^6 PFU/ml) every 12 h and 10^6 PFU intrarectally via suppository	During the 11 months of follow-up no *K. pneumoniae* was isolated, however, other infections were reported	[59]
63-year-old, female	Chronic UTI	XDR	Two cocktails: SZ-1, SZ-2, SZ-3, SZ-6 and SZ-8; and Kp152, K154, Kp155, Kp164, Kp6377, and HD001	Bladder irrigation of phages (5x10^8 PFU/ml) once a day during 5 days combined with oral trimethoprim-sulfamethoxazole	The patient was discharged home after less than one month from phage treatment and a follow-up of 6 months found no recurrence	[60]
60-year-old male	Recurrent UTI	MDR (variable susceptibility to carbapenems and full susceptibility to colistin only)	Phages from the Experimental Therapy of the Polish Academy of Science	Intrarectal application of 10 ml of phages twice daily combined with meropenem	After 18 d of combined treatment, less symptoms were observed but recurrence ended in resection of the infected organ	[61]
58-year-old, male	Recurrent UTI and epididymitis	ESBL susceptible to meropenem and amikacin	Phages from Eliava Institute (Tbilisi)	Two months with 2 oral vials per day and one via intravesical every second day	No bacteria was isolated after 14 months of follow-up	[62]
40-year-old, male	Lung infection	Pandrug-resistant	KPV811 and KPV15	Four days of 2 ml of inhaled phages and 18 ml of nasogastric administration (1x10^8 PFU/ml). Two days once a day and two days twice a day. Ceftazidime, linezolid, colistin, meropenem, cotrimoxazole and tobramycin were also administered	*K. pneumoniae* susceptible to antibiotics was detected after phage therapy only in stool samples, not in bronchial lavage	[63]

Abbreviations: ESBL, extended-spectrum beta-lactamase; IV, intravenous; MDR, multidrug resistant; PFU, plaque-forming units; XDR, extensively drug resistant.

Table S3. Summary of case reports using phage therapy against *P. aeruginosa* in patients from 2015 to date.

Age & Gender	Type of Infection	Resistance	Bacteriophage	Phage Therapy	Key Clinical Points & Outcome	References
61-year-old, male	Acute kidney damage and sepsis	MDR only sensitive to colistin	Phage cocktail BFC1 with 2 phages	IV administration of phage therapy every 6 h for 10 d and phage cocktail irrigation in his wounds every 8 h for 10 d	After phage therapy administration, blood cultures turned negative, and kidney was successfully fully recovered	[91]
2-year-old, male	DiGeorge syndrome, complex congenital heart disease and recalcitrant bacteraemia	MDR	Cocktail containing two phages	Phage cocktail administration at a dose of $3.5x10^5$ PFU/ml every 6 h	The patient exhibited adverse reaction or resistance to multiple antibiotics (meropenem, tobramycin, aztreonam, colistin and polymyxin B) to which the organism was previously susceptible. Patient tolerated first 6 doses of phage therapy but after that phage administration was suspended due to anaphylaxis attributed to progressive heart failure. Once the patient returned phage therapy, blood cultures turned negative coinciding with clinical improvement	[92]
80-year-old, female	Diabetes mellitus type 2, chronic kidney failure and relapsing right knee periprosthetic joint infection	Two different strains of *P. aeruginosa* (one colistin-only sensitive and other only susceptible to colistin and ceftazidime)	Unknown bacteriophage	Bacteriophage solution (10^8 PFU/ml) was applied locally (during surgery) and IV every 8 h for 5 d combined with antibiotic treatment: colistin (150 mg every 24 h), meropenem (1 g every 12 h) and ceftazidime (2 g every 12 h)	After combined treatment with bacteriophages and antibiotics, cultures from drainage fluid turned negative	[93]
29-year-old, female	CF with pulmonary exacerbation that led to acute-on-chronic respiratory failure due to infection	Two strains of *P. aeruginosa* (one only sensitive to colistin and other sensitive to meropenem and piperacillin / tazobactam)	Phage cocktail AB-PA01 (contains 4 lytic phages)	IV administration of cocktail ($4x10^9$) every 6 h for 8 weeks. During first 3 weeks, phage therapy was combined with administration of ciprofloxacin and piperacillin / tazobactam. For the last portion of phage therapy, ciprofloxacin was discontinued and changed for doripenem	After 100 d since bacteriophage was finished, the patient did not suffer recurrence infections and CF exacerbation and even, 9 months later, she underwent successful bilateral lung transplantation	[94]
67-year-old, male	Bilateral lung transplantation for hypersensitivity pneumonitis. Post-transplant course was complicated due to multiple medical issues, including two different episodes of pneumonia	Two different strains of *P. aeruginosa* in two different episodes (one only sensitive to amikacin and tobramycin and the other also sensitive to piperacillin / tazobactam). Both strains were intermediate to colistin	Phage cocktail AB-PA01 (contains 4 lytic bacteriophages), AB-PA01-m1 (AB-PA01 plus one new specific phage) and NAVY phage cocktail (personalized phage cocktail)	During first episode of infection, 2-week IV and nebulized phage therapy AB-PA01 ($4x10^9$ PFU/ml) combined with antibiotic treatment (piperacillin / tazobactam and colistin) was performed. While during second episode infection, physicians performed a combined treatment with two different bacteriophages: AB-PA01-m1 ($5x10^9$ PFU/ml) and NAVY phage cocktail ($1x10^9$ PFU/ml); with antibiotics (piperacillin / tazobactam, tobramycin and inhlated colistin)	After first combined treatment with bacteriophages and antibiotics, cultures did not show bacterial burden. However, one month after combined treatment was finished, patient's clinical conditions worsened, and respiratory cultures turned positive. Once the second combined treatment was employed, clinical resolution of pneumonias was observed	[95]

57-year-old, female	Bronchiectasis colonized with *PA* causing recurrent infections.	MDR only sensitive to colistine	Phage cocktail AB-PA01 (contains 4 lytic bacteriophages)	4-week IV administration of phage therapy (4x10^9 PFU/ml) combined with inhaled colistin	Since start to the end of combined treatment, no additional *Pseudomonas* was cultured from respiratory samples	[95]
77-year-old, female	Posterolateral mini-thoracotomy for resection of right lower lobe adenocarcinoma with mediastinal node sampling developing pneumonia and empyema	Strain sensitive to piperacillin-tazobactam, ciprofloxacin and meropenem	Phage cocktail AB-PA01 (contains 4 lytic bacteriophages)	Twice-daily intravenous and nebulized administration bacteriophages (1x10^9 PFU/mL) combined with antibiotics (IV administration of ciprofloxacin and gentamicin)	Since day 4 until 6 months after completation of combined therapy, the patient remained culture-negative	[96]
13-year-old, male	Infected thoracotomy wound after double lung transplantation in a CF patient	MDR	Bacteriophages PA5 and PA10	Locally administration, intraoperatively mixed with fibrin glue, of bacteriophages combined with intravenous administration of antibiotics: colistin (twice per day), ceftazidime (750 mg twice per day) and avibactam (187.5 mg three times per day)	The wound completely healed and *P. aeruginosa* was not detected after combined treatment with bacteriophages and antibiotics	[63]
76-year-old, male	Aortic arch replacement surgery with Dacron graft for an aortic aneurysm developing recurrent infections.	Resistance associated to biofilm	Bacteriophage OMKO1	Bacteriophage administration (10^7 PFU/ml) was proved as an adjuvant to ceftazidime antibiotic treatment (0.2 g/ml)	The patient did not manifest any evidence of recurrent infection remaining off antibiotics	[97]

Abbreviations: CF, cystic fibrosis; IV, intravenous; MDR, multi-drug resistant; PFU, plaque-forming units.
